# Genetic Regulation of Transcription in the Endometrium in Health and Disease

**DOI:** 10.3389/frph.2021.795464

**Published:** 2022-01-03

**Authors:** Sally Mortlock, Brett McKinnon, Grant W. Montgomery

**Affiliations:** Institute for Molecular Bioscience, The University of Queensland, Brisbane, QLD, Australia

**Keywords:** endometrium, transcription, gene expression, disease, genetic regulation, hormones, menstrual cycle

## Abstract

The endometrium is a complex and dynamic tissue essential for fertility and implicated in many reproductive disorders. The tissue consists of glandular epithelium and vascularised stroma and is unique because it is constantly shed and regrown with each menstrual cycle, generating up to 10 mm of new mucosa. Consequently, there are marked changes in cell composition and gene expression across the menstrual cycle. Recent evidence shows expression of many genes is influenced by genetic variation between individuals. We and others have reported evidence for genetic effects on hundreds of genes in endometrium. The genetic factors influencing endometrial gene expression are highly correlated with the genetic effects on expression in other reproductive (e.g., in uterus and ovary) and digestive tissues (e.g., salivary gland and stomach), supporting a shared genetic regulation of gene expression in biologically similar tissues. There is also increasing evidence for cell specific genetic effects for some genes. Sample size for studies in endometrium are modest and results from the larger studies of gene expression in blood report genetic effects for a much higher proportion of genes than currently reported for endometrium. There is also emerging evidence for the importance of genetic variation on RNA splicing. Gene mapping studies for common disease, including diseases associated with endometrium, show most variation maps to intergenic regulatory regions. It is likely that genetic risk factors for disease function through modifying the program of cell specific gene expression. The emerging evidence from our gene mapping studies coupled with tissue specific studies, and the GTEx, eQTLGen and EpiMap projects, show we need to expand our understanding of the complex regulation of gene expression. These data also help to link disease genetic risk factors to specific target genes. Combining our data on genetic regulation of gene expression in endometrium, and cell types within the endometrium with gene mapping data for endometriosis and related diseases is beginning to uncover the specific genes and pathways responsible for increased risk of these diseases.

## Introduction

Human endometrium lines the inner surface of the uterus and plays a vital role in female reproduction and maintenance of pregnancy, providing the receptive microenvironment for embryo implantation and placental development. Endometrium is composed of several cell types including luminal and glandular epithelial cells, endometrial stromal cells, vascular cells and immune cells (1). In preparation for embryo implantation endometrial stromal fibroblasts (ESCs) terminally differentiate to secretory decidual stromal fibroblast cells (DSCs) and in the absence of conception the tissue undergoes controlled shedding, tissue repair, re-epithelialisation, regeneration and remodelling (2). This process is cyclical, averaging 25–30 days in length, and is controlled by ovarian steroid hormones (2, 3). During the menstrual cycle the endometrium is continuously undergoing cellular proliferation, differentiation and structural remodelling in response to circulating steroid hormones. These changes in cellular function and composition reflect the changing roles of this dynamic tissue and can be broadly defined into stages of endometrial development. An initial proliferative phase is characterised by endometrial tissue regeneration and cellular proliferation that prepares for embryo implantation and preceds ovulation. It is followed by the secretory phase with development of more complex glands, spiral arteries and stromal oedema designed to support a developing embryo in response to progesterone secreted by the corpus luteum (4, 5). In the absence of pregnancy the functional layer is shed during the menstrual phase before repair and regeneration commences again (1).

**Figure 1:**
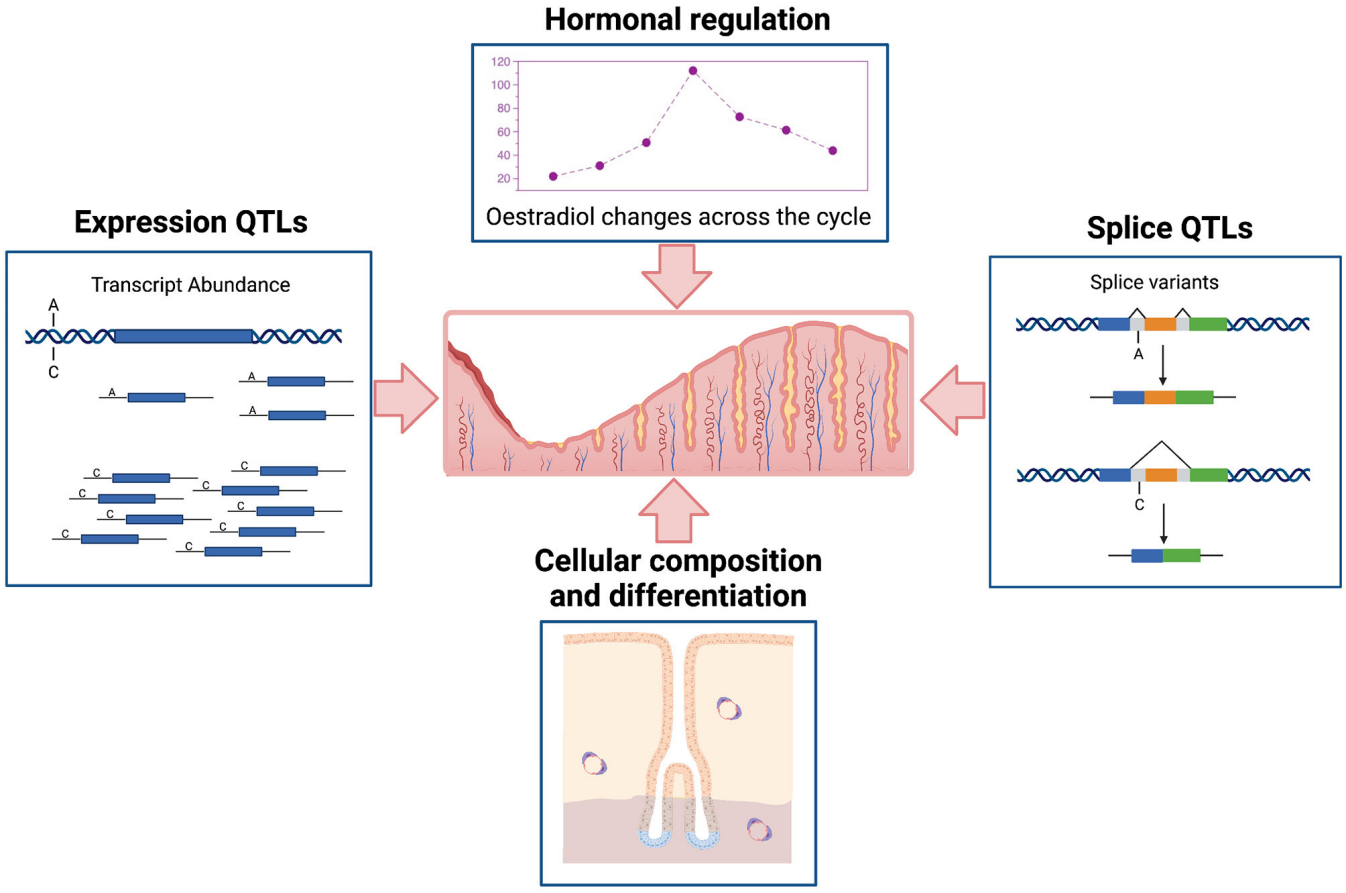
Schematic representation of major factors affecting variation in transcription in human endometrium. Created with BioRender.com.

It is important to understand the complex regulatory processes influencing gene expression in the endometrium and relationships to endometrial structure and function, fertility, and reproductive pathologies. Gene expression in the endometrium is dominated by events across the menstrual cycle and influenced by hormonal regulation and changing cellular composition (Figure 1). Patterns of expression for individual genes show marked variation (6, 7), with expression of some genes high in the proliferative phase and then decreasing in the secretory phase or the reverse patten with low expression in the proliferative phase and increasing later during the secretory phase. Some genes are on for only a few days and others show variable patterns of expression detected only in a proportion of individuals (7). In addition to the cycle changes observed in most individuals, epigenetic signatures (8, 9) and the expression of many genes is under genetic control in endometrium (Figure 1) and other tissues contributing to variation between individuals (6, 7, 10).

The purpose of this review is to discuss the regulation of gene expression in endometrium. It is not intended to provide a systematic review of the endometrial gene expression literature, but to review major factors influencing the expression of genes in the endometrium and lessons from recent studies in other tissues.

## Hormone Regulated Endometrial Gene Expression

The complex cellular anatomy and diverse functions of human endometrium are reflected in the dynamic nature of endometrial gene expression. Analysis of gene expression measured in eutopic endometrial samples using microarray and RNA-seq technologies has shown significant differences in the expression of thousands of genes across the menstrual cycle (6, 7, 11–14). Studies show that these differences in gene expression occur both at the level of mean expression and differences in gene activation i.e., the proportion of samples expressing individual genes. An average of 62–66% of genes expressed in endometrium were expressed in >90% of samples however, the remaining 34–38% of genes were transcribed in varying proportions of samples (6, 7). Evidence suggests that the expression of more than 30% of genes in the endometrium differ significantly in mean expression or in the proportion of samples expressing each gene across the menstrual cycle. These genes are enriched in hormone response pathways, transcription factor targets and epithelial mesenchymal transition pathway (6).

The biggest differences in gene expression are observed between the proliferative and secretory phases of the menstrual cycle and between stages (early, mid and late) within the secretory phase of the cycle (6, 12). Transition between the proliferative to the early secretory stages has been characterised by the upregulation of genes involved in metabolic processes, negative regulation of cell proliferation, hormone response and secretion. Down-regulated genes are enriched in cell cycle regulation and cellular mitosis and division pathways (6, 12, 13). Subtle changes have also been reported within the proliferative phase which is characterised by healing and cell proliferation (6, 15). Genes upregulated in the proliferative phase have roles related to cell proliferation, differentiation, tissue remodelling, immunomodulation and angiogenesis (14, 15). Differences in expression between the early and mid-secretary phases likely reflect the cellular and molecular events governing endometrial receptivity and preparation for implantation. Upregulated genes are involved in cell adhesion, motility and communication, growth factor and cytokine binding and signalling, the immune and inflammatory responses and hormone response. Down-regulated genes are involved in cell division (6, 13, 14). Finally the transition between the receptive mid-secretary to late-secretary phase is characterised by preparation of the tissue for desquamation and menstruation reflected in changes in expression of genes involved in alterations of the extracellular matrix, the cytoskeleton, cell motility, communication and adhesion, vasoconstriction, immune response, wound healing and inflammatory mediation (6, 13, 14).

Menstrual cycle phase is a major source of variability in endometrial datasets and consideration must be given to apply appropriate corrections for cycle phase when analysing data generated from endometrial samples. Observed changes in gene expression across the menstrual cycle are likely mediated by a combination of changes in cell composition and response to changing levels of circulating hormones. The expression of some genes in the endometrium is reported to change in response to fluctuating levels of steroid hormones oestrogen and progesterone (12, 13, 16, 17). Response to circulating hormones, oestrogen and progesterone, is mediated through several hormone responsive genes, regulators and mediators and has been reviewed in detail elsewhere (1, 18). Oestrogen receptor (*ESR1*) and progesterone receptor (*PGR*) have been shown to be vital to maintaining healthy gene regulatory networks in the endometrium (18). *PGR* plays an important role regulating cell differentiation and proliferation through extracellular signal-regulated kinase/mitogen-activated protein kinase (ERK/MAPK) and Protein Kinase B (AKT) pathways, and its target genes (e.g., *IHH, HOXA10, IGFBP1, STAT3, FOXO1, SOX17*) are required for successful implantation and decidualisation (18, 19). *ESR1* regulates endometrial epithelial proliferation, promotes stromal cell differentiation and is critical for endometrial receptivity and decidualisation through its induction of cytokines, IGF1 signalling, Wnt/β-catenin signalling, FGF signalling, ERK-MAPK signalling and PGR signalling (18, 20). The importance of hormonally driven regulatory pathways in healthy endometrial function is reflected by their dysregulation in endometrial pathologies including endometrial cancer (21) and endometriosis (18). However, not all genes with variable expression across the menstrual cycle are correlated with hormone levels or hormone receptors (*ESR1, PGR*) suggesting hormonal regulation alone cannot explain all the variation in gene expression (6, 22). Inter-individual variation in gene expression not explained by menstrual cycle stage is likely the result of a combination of cell type composition, genetic regulation and environmental effects.

Regulation of genes in the endometrium plays a vital role in female fertility. The Human Gene Expression Endometrial Receptivity database (HGEx-ERdb) is a compilation of datasets that provides information about the expression of >19 k genes in human endometrium during various phases of the menstrual cycle and in other conditions (23). Genes with consistent patterns of differential expression in endometrium during the receptive phase have been classified as receptivity associated genes (RAGs). RAGs play a role in the regulation of pathways that facilitate the structural and functional modifications required for successful embryo implantation (23). Several RAGs have been identified as potential biomarkers for endometrial receptivity (23–25). Biomarkers for endometrial receptivity can be used as diagnostic tools in reproductive medicine. One such tool, the endometrial receptivity array (ERA), can be used to diagnose receptivity in women with recurrent implantation failure (RIF) to guide decisions around personalised embryo transfer as a therapy (26). Receptivity signatures continue to be refined further to define different transcriptomic signatures within the receptive phase that are associated with clinical outcomes such as successful pregnancy (27). There are also extensive changes in endometrial gene expression profiles in response to embryonic signals and physiological changes during pregnancy (28, 29). *In vitro* studies in human endometrial stromal cells have shown changes in expression of thousands of genes in response to trophoblast cells including induction of immune and angiogenic pathways (30, 31).

## Genetic Regulation of Transcription

### Expression Levels

**Figure 2:**
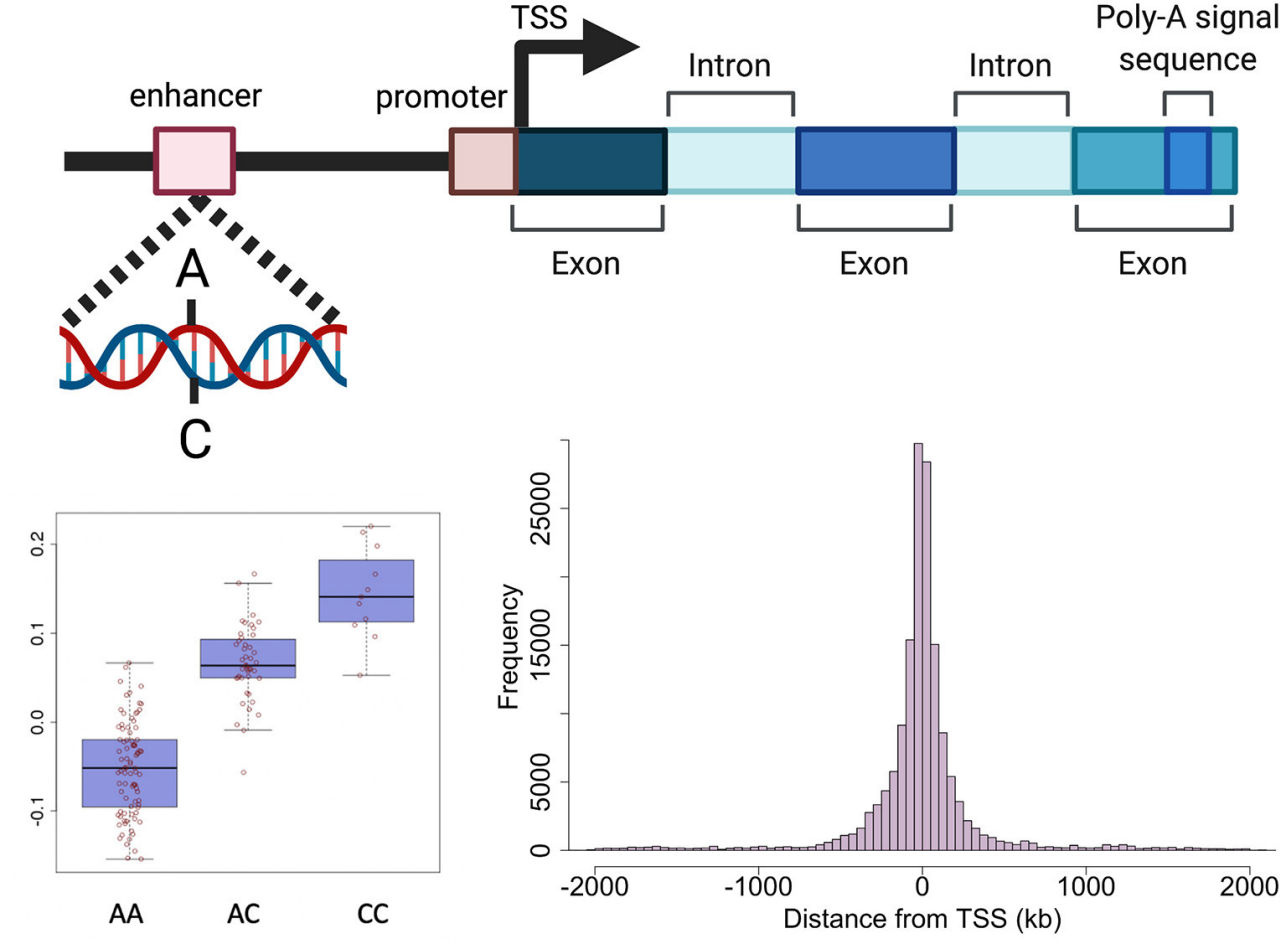
Schematic representation of genetic regulation of gene expression. Expression quantitative trait loci (eQTL) describe the association between genetic variation and variation in the level of gene expression. The top panel depicts how a genetic variant located in a regulatory region (e.g., enhancer) can effect the level of expression of a nearby gene. The presence of a particular allele can be associated with an increase or decrease in expression of the gene as shown in the boxplot in the bottom left panel. The histogram in the bottom right panel shows the distance between variants effecting gene expression and the target gene in human endometrium. Created with BioRender.com.

Genetic variants can effect transcription through various mechanisms including, but not limited to, altering promoters, transcription factor (TF) binding sites, enhancers, regulatory ncRNAs, RNA splicing and UTRs (important for post-translational regulation) (32). The expression of a large proportion (>80%) of genes expressed in tissues is regulated by genetic variation defined as expression quantitative trait loci (eQTLs) (10, 33). An eQTL denotes the association between a genetic variant (eSNP) and expression levels of mRNA transcripts of either a nearby gene (*cis*) or distant gene (*trans*) (Figure 2). cis-eQTLs are commonly located close to transcription sites (Figure 2) with 70% of eSNPs within 300 kb of the gene transcription start sites (6, 10, 34, 35). Consortium efforts have generated large eQTL datasets in multiple human tissues including 49 tissues from GTEx (33) and in blood (10). The majority of eQTLs (>70%) observed in smaller eQTL studies are shared between tissues. The recent large eQTL meta-analysis for blood (eQTLGen) derived gene expression analysis from 31,684 individuals and identified *cis*-eQTLs for 88% of autosomal genes expressed in blood (10). The replication rate for these eQTL in GTEx tissues excluding blood was much lower (15%) than previous studies. This may be a power issue, but could suggest eQTLs with smaller effects may be more tissue specific. The median pairwise correlation of eQTL effect sizes (r_b_) between tissues is estimated as 0.55 across GTEx tissues and higher within biologically similar tissues such as skin tissues (r_b_ = 0.80), arterial tissues (r_b_ =0.74) (36, 37) and between brain tissues (r_b_=0.94) (38).

*Cis*-eQTLs for 627 genes have been identified in endometrium using microarray and RNA-seq technologies (6, 7, 13). The most recent endometrial eQTL study was conducted using RNA-seq data generated from the endometrial samples of 206 European women identifying significant genetic effects on the expression of 444 genes (7). Mapping of endometrial eQTLs was performed independent of menstrual cycle phase, with cycle phase included as a covariate in the analysis. Compared to larger eQTL datasets like eQTLGen which identified >6,000 *trans*-eQTL genes, smaller endometrial eQTL datasets have had limited power to detect distal genetic effects on gene expression in the form of *trans*-eQTLs. *Trans*-eQTLs for only 28 genes have been validated between studies (6, 7). Subsequent context specific analyses did not detect any eQTLs with effects that differed between menstrual cycle phases or pathologies. Importantly, several genes used to assess endometrial receptivity on the ERA had evidence of genetic regulation suggesting an individual's genetic background may also influence the appropriate time for embryo transfer (6).

The majority (85%) of cis-eQTLs in endometrium were also reported in other tissues including ~72% detected in blood however, eQTLs for 61 genes appear to be specific to endometrium (7). Endometrial eQTLs were highly correlated with other reproductive tissues such as ovary and uterus (7). Interestingly, the effects of eQTLs in endometrium were also highly correlated with digestive tissues (r_b_ > 0.67), possibly reflecting similarities in tissue structure, cell composition, and functions between the tissues (7). Overlap in expression profiles and genetic regulation between endometrium and other tissues may underpin some comorbid relationships between endometrial disorders and other diseases through shared genetic risk loci. Studies have reported epidemiological associations and genetic correlation between subfertility and gastrointestinal disease (39) and between endometriosis and abnormalities in gastric mucosa (40, 41), uterine fibroids (42) and ovarian cancer (43).

### Splicing

Genetic variants can also regulate splicing of mRNA transcripts in addition to regulation of gene expression level. Splicing is a process whereby pre-mRNA is spliced at different sites to produce multiple mRNA isoforms that include or exclude different exonic sequences (44). Alternative splicing (AS) has been estimated to occur in 95–100% of human mRNA that contain >1 exon (45). Comparisons of AS between tissues have shown that 47–74% of splicing events show variation between tissues and 10–30% show individual-specific variation (46). The ability to map transcripts using RNA-seq data and correlate splicing events with genetic variants has allowed identification of splicing quantitative trait loci (sQTLs) (47, 48). *Cis*-sQTLs have been identified in multiple tissues in the GTEx data, 210,485 sQTLs affecting 6,963 genes (sGenes) were identified across 48 tissues averaging 1,158 genes per tissue (49). Overall 44% of protein coding genes had an sQTL compared to eQTLs identified in 95% of protein coding genes (49). In the GTEx data, there is a high correlation of sQTL effect sizes between biologically similar tissues and overall 66% of sGenes are shared between tissues, similar to that observed for eQTLs (49). sQTL sharing analysis has shown that reproductive tissues (uterus, ovary, vagina) cluster together alongside arterial and gastrointestinal tissues. eQTLs are only observed for 52% of genes with sQTLs and in particular, tissue specific sQTLs do not necessarily have tissue-specific gene level expression highlighting the importance of characterising regulation of gene expression at the different levels and in relevant tissues (49). To date there has been no comprehensive analysis of sQTLs in endometrial tissue. Data available from GTEx shows a total of 182,070 sQTLs have been identified in uterus for 12,800 sGenes, 94% of these sGenes also had sQTLs in ovary or vagina compared to 66% in blood. sQTL mapping in endometrium using available RNA-seq and genotype data is ongoing.

### Methylation

Epigenetic mechanisms such as DNA methylation can regulate transcription by recruiting methyl-CpG-binding proteins involved in gene repression or by inhibiting the binding of specific transcription factors (50). Variation in DNA methylation (DNAm) profiles in the endometrium across the menstrual cycle have been reported (8, 51–53), although these changes are less marked than observed changes in gene expression. Differentially methylated sites have been shown to correlate with expression of nearby genes in endometrium and are enriched for genes also reported as differentially expressed across the menstrual cycle (8, 51, 52). Genetic variation has been associated with methylation at 4,546 CpG sites in endometrium, defined as methylation quantitative trait loci (mQTLs) (8). 414 endometrial mQTLs were associated with the expression of 186 genes suggesting genetic regulation of gene expression in endometrium can also be mediated through methylation. Potential endometrial specific mQTLs have been annotated to genes with roles in hormone responsive proliferation, maintenance of cell structural integrity and adhesion and endometrial receptivity (8). Genetic regulation of methylation in endometrium has also been associated with reproductive traits including endometriosis, age at menopause and ovarian cancer (8). One example on chromosome 2 features a variant (rs11674184) associated with both endometriosis and methylation at cg16908938, a CpG site located in an intron of *GREB1* (8). This same variant is associated with alternative splicing of *GREB1* in GTEx data (49).

## Cell Specific Regulation of Transcription

**Figure 3:**
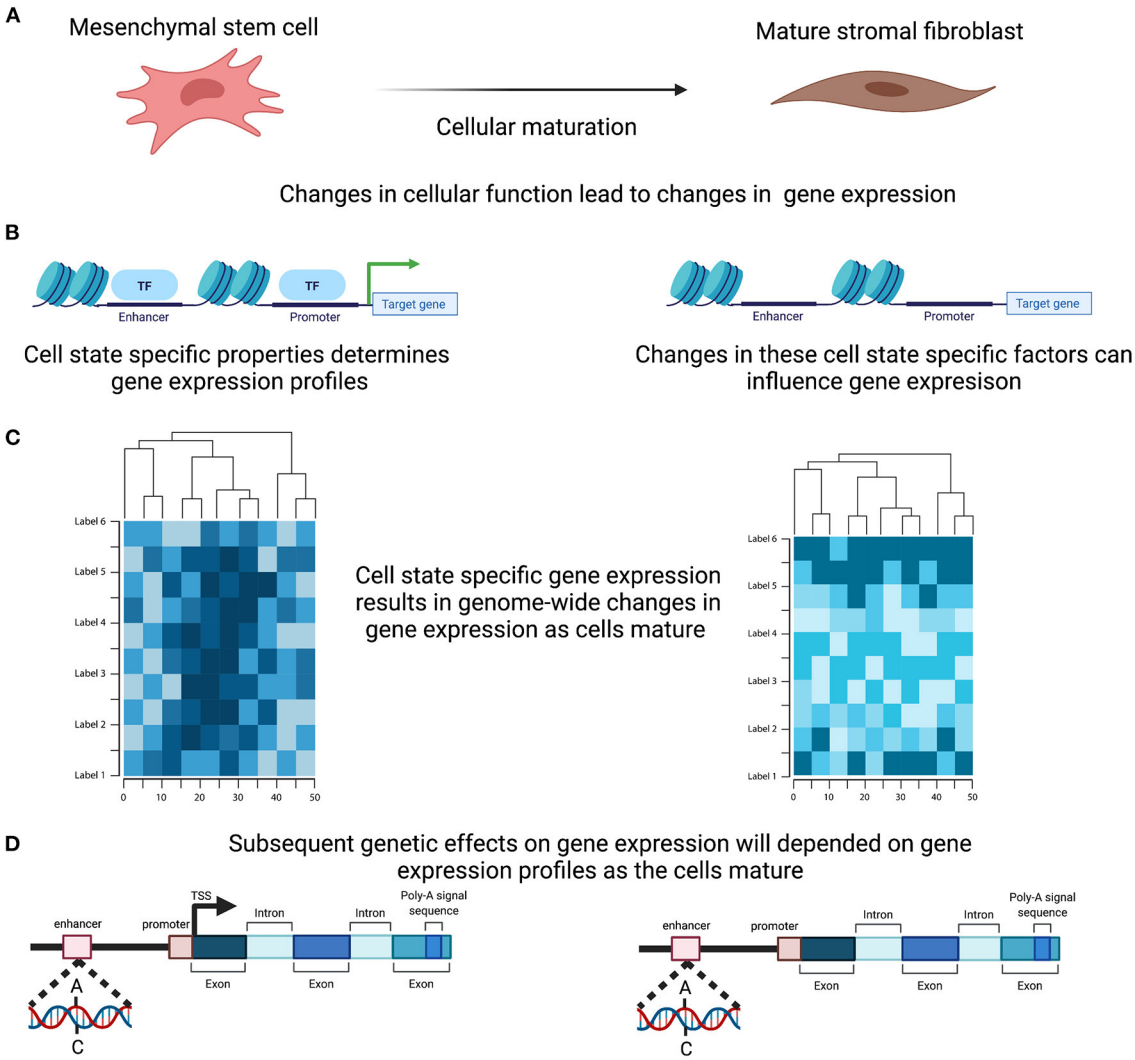
Cell Specific genetic effects on gene expression. **(A)** Cells within the endometrium, such as the mesenchymal stem cells, undergo proliferation and maturation each menstrual cycle to generate a mature cell. **(B)** Maturation is controlled by a programmed course of gene expression that results in cell specific properties and are reflected in alteration in the active transcription. **(C)** In combination and across the genome these active change in expression will induce distinct genome wide gene expression profiles. **(D)** Genetic effects on transcription therefore will ultimately induce differing effects as the cells mature. Created with BioRender.com.

The functions of complex tissues, such as the endometrium, are facilitated by the interaction of multiple cells with divergent roles. Each cell has a unique life cycle from maturation to programmed cell death and are often categorised based on their developmental pathway, degree of maturation and resulting form and function, all of which are driven by a programmed course of gene expression that will underpin their individual role within the tissue. Expression levels for each gene in the endometrium therefore reflects not only the range and proportions of different cell types present within the sample but also their degree of differentiation, maturation and current state of activation (Figure 3). Genetic regulation of gene expression occurs in a cell type specific manner (54–56) and evidence is emerging that this is also the case with cell state (57). Characterising the changing cellular composition, as well as the developing cellular state and identifying how this interacts with the genetic influence on gene expression will be required to understand endometrial transcription, endometrial function and how perturbations in this mechanism contribute to endometrial disease susceptibility (Figure 3).

The endometrium functions through contributions from epithelial, stromal, immune and vascular cells. The dynamic nature of the tissue means that the relative composition and state of these cells is in a constant state of flux. Endometrial stromal and epithelial cells form the majority of the 5–7 mm thick multi-functional tissue (58). In response to changing progesterone concentrations endometrial stromal cells undergo transcriptional reprogramming, potentially through the regulation of promyelocytic leukaemia zinc finger (PLZF) to drive decidualisation, altering both gene expression signature and consequent function (59). The process of decidualisation encompasses a complex network of regulatory processes involving hormonal, biochemical, immunological and molecular factors recently reviewed by Ng et al. (60). Important molecular regulators of decidualization include Homeobox A10 (*HOXA10*), Wnt Family Member 4 (*WNT4*) and Forkhead box O1 (*FOXO1*) which targets transcription of decidual genes Prolactin (*PRL*) and insulin-like growth factor-binding protein 1 (*IGFBP1*) (60–62). Similarly, epithelial cells can be directed toward luminal, or glandular, ciliated or non-ciliated epithelial cells, mediated by divergent differentiation (63). Both tissue resident immune cells and immune cells that are derived from the transient infiltration from systemic circulation in response to changes in oestrogen and progesterone concentrations (64) will also influence endometrial cellular composition. Endometrial regeneration is also accompanied by a restoration of vascular integrity and angiogenesis (65), expanding the endothelial component. Cellular composition and the proportions of different cell types can be altered significantly by different pathologies (66).

The dynamic nature of the endometrial tissue means that these divergent cell types also present with a broad continuum of cell states. Endometrial regeneration is initiated from just a few endometrial mesenchymal stem and epithelial progenitor cells that remain in the basalis layer after menstruation (67–69). The eMSC are derived from perivascular locations (69) representing a subset of CD140+/CD146+ pericytes (70) with a gene expression signature that is highly dependent on the microenvironment (71) and may contribute essential cytokines and growth factors to the stem cell niche (72). Additionally, within this niche there is emerging evidence of the presence of additional pluripotent, very small embryonic-like (VSEL) stem cells, identified in reproductive tissue of humans such as testis (73), as well as the endometrium of mice that may contribute to the regeneration of functional endometrium, as well as the regeneration of their damaged endometrial tissue (72).

In response to secreted molecules and extracellular matrix signals from the stem cell niche environment (74), regulatory changes are initiated that facilitate gene activation and stimulate cellular differentiation toward a cascading progression of cell states. Differentiation and maturation of each cell from their progenitor is therefore, both driven and accompanied by, changes in their gene expression profile. Genetic variants that influence the ability of these regulatory mechanisms of gene expression, through the mechanisms discussed above, will have significant influence on the transcriptomic signatures, timing of cellular maturation and may contribute to subtle variations that lead to endometrial pathologies. Better understanding the interaction between genetic variants and cellular development will be vital to understand the regulation of gene transcription in tissues.

Unravelling the composition and contribution of individual cells within complex tissue is however challenging. To study the genetic influence on cells individually requires the ability to focus on individual cells within a complex mixture. For endometrium we are yet to establish a comprehensive cell atlas to examine cell composition across the changing stages of the menstrual cycle. Although admiral efforts are being made these still require more than single patients to capture the natural variability of the population (75). Using whole excised endometrial tissue inhibits the possibility of directly studying the contribution of each cell to the tissue gene expression signature. Additionally, the site of tissue biopsy may contribute to variation in the cellular composition of each sample and/or variation between individual participants in a study. Conversely, physical dissociation of individual cell types and subsequent gene expression analysis result in the removal of the niche environmental factors that modulate cell state and its associated gene expression signatures. It is also a labour intensive process that requires significant work to collect the sample numbers required for studying the effects mediated by genetic regulation. A number of experimental designs and novel techniques are however being utilised to overcome these challenges.

### Single-Cell Transcriptomics

Single-cell RNA-sequencing offers a new avenue to investigate cellular heterogeneity of endometrial tissue and assess the influence of genetic variants in individual cells and cell types. Early studies using single-cell transcriptomics to profile endometrial cell populations across the menstrual cycle identified seven main cell types based on clustering and expression of canonical markers and differentially expressed genes. These cell types included stromal fibroblasts, endothelial cells, macrophages, lymphocytes, ciliated and unciliated epithelial cells and smooth muscle cells with mesenchymal stem cell characteristics (75). Different expression profiles within each cell type were detected across the cycle including epithelial and stromal profiles likely to characterise the transition between early and late proliferative and early secretory endometrium, as well as the transition into the window of implantation with the upregulation of known receptivity genes (75). Sub-populations of stromal and epithelial cells have also been identified (76), some of which have been associated with endometrial pathologies (77, 78). Ma et al. (78) observed that characteristics of the eutopic endometrium between women with and without endometriosis were generally similar, however there was evidence of differences in the cell subtypes reflected by gene expression (78), suggesting that some inconsistencies in observed differences in gene transcription in bulk endometrial tissue may be confounded by cellular heterogeneity. Single-cell transcriptomic studies in endometrium have been limited by small sample sizes and the type of endometrial sample used which may not fully capture all relevant endometrial cell types.

Combining single-cell transcriptomic data with genome-wide genotyping information also provides the opportunity to assess the genetic regulation of gene expression in individual cells, cell types and the impact on changes in cell state. Recent evidence in skin fibroblasts from 79 donors found the majority of eQTLs were specific to cell subtypes and reprogramming these cells into iPSC resulted in almost all eQTL disappearing entirely (57), suggesting genetic regulation is dynamic across both individual cells and during cell maturation. This is an emerging opportunity to be investigated in endometrial tissue. While providing powerful new insights, the depth of sequencing produced by single cell sequencing limits the data to only the highly expressed genes, often missing genes that only require low expression to initiate cascading events, thereby limiting the potential to identify important changes. Both the 5' and 3' amplification procedure also limits the potential to examine the genetic influence on splicing. Techniques that can perform high throughput are yet to be developed.

### Bioinformatic Deconvolution and Cell Type Enrichment

Computational methods have been developed in the last 10 years to dissect cellular heterogeneity and account for its influence on tissue gene expression profiles (66, 79, 80). Two main approaches include, deconvoluting cellular composition and enumeration of cell subsets, and assessing the enrichment of indivual cell types. These methods use additional data from a “reference” gene set from purified cell types or single cell RNA sequencing to define cell-type specific gene signiatures. Computational methods to deconvolute bulk gene expression data enable some cell type specific inferences, but their accuracy depends on the availability of expression profiles for relevant cell types. In addition, several sources of variation influence the use and interpretation of methods of cellular decomposition of bulk RNA-seq data containing mixed cell types (81). The performance of deconvolution methods varies with cell type, source laboratory and tissue. The methods are strongly influenced when individual cell profiles and mixed tissue sample originate from different laboratories and when profiles are generated from single cell sequencing (81). Careful consideration is needed when applying these approaches to endometrial data without well-characterised endometrial cell type specific gene signatures. Studies are underway to dissect the cellular heterogeneity in endometrium by adapting existing approaches and incorporating signatures from endometrial cell-types using sorted cell expression data and single-cell RNA-seq (82).

### Multiplexing Spatial Transcriptomics

Spatial transcriptomics offers the potential to identify transcriptome expression at a single cell level while maintaining spatial resolution and capturing the niche influences. Currently it is limited because of the expense for individual slides. However, methods are being developed that will allow multiple samples to be included on individual slides. Accompanying this data with genome-wide genotype information will map changes in cell state associated with changes in genetic regulation.

## Association Between Genetic Risk Factors for Disease and Transcription

Gene expression is an indicator of cellular state and misregulation of gene expression can be indicative of disease. Gene expression signatures in the endometrium have been associated with endometrial traits and disease. Evaluation of gene expression in the receptive phase has identified signatures for recurrent implantation failure characterised by downregulation of genes involved in cell cycle regulation and cell division and cytoskeleton and cilia formation (83). Obesity has also been associated with significant transcriptional changes during window of implantation (WOI) which may contribute to lower implation rates seen in obese women (84). Transcriptional dysregulation in proliferative-to-secretory transition and during the WOI in endometrium of women with moderate/severe endometriosis has also been reported (85, 86). Differences in gene expression in eutopic endometrium between women with and without endometriosis have also been reported however, candidate endometriosis susceptibility genes have failed to replicate between studies. In larger studies with greater power, significant differences in expression between women with and without endometriosis are not detected following correction for menstrual cycle stage and appropriate correction for testing multiple gene signatures (6, 7, 53). Allowing for variation induced by the individual genetic background could explain and be applied to reduce the inconsistencies.

Genetic variants regulating transcription in endometrium have been associated with several reproductive traits and diseases. SNPs regulating gene expression have been associated with age at menopause, age at menarche, endometriosis, polycystic ovarian syndrome (PCOS), endometrial cancer and ovarian cancer (6, 7). Formal statistical tests should be used to determine if the same causal SNP effects both endometrial gene expression and the disease trait. Methods include Bayesian colocalization analyses such as COLOC (87) and transcriptome-wide association analyses (TWAS) such as Summary-data-based Mendelian Randomisation (SMR) (88), PrediXcan (89) and TWAS-Fusion (90).

Transcriptome-wide association analyses (TWAS) assess the association between the expression of each gene and a trait. In the absence of gene expression data from a large sample the expression of genes can be predicted using eQTL information or a reference set containing expression data and its association with genetic variants (91). SMR is a Mendelian randomisation approach that integrates eQTL and GWAS summary statistics to identify associations between gene expression and complex traits and also applies a heterogeneity test to distinguish pleiotropy from linkage (88). Endometrial eQTLs have been used in SMR analyses alongside summary statistics from a range of reproductive traits and diseases identifying several genetic variants regulating both expression of genes in endometrium and traits. Integration of GWA summary statistics for endometriosis (92) and endometrial eQTLs using SMR identified significant associations between the expression of three genes in endometrium, Long Intergenic Non-Protein Coding RNA 339 (*LINC00339)*, Vezatin (*VEZT)* and FYVE, RhoGEF And PH Domain Containing 6 (*FGD6)* and risk of endometriosis (6, 7, 93). Given the large overlap of eQTLs between endometrium and blood, large blood eQTL datasets can also be used as a proxy for genetic regulation of expression in endometrium, such approaches identifying association between genetic regulation of expression of *VEZT*, Cell Division Cycle 42 (*CDC42), LINC00339* and endometriosis (7, 92). Similar analyses identified genetic regulation of transcription in endometrium is associated with other reproductive traits and pathologies including expression of Neighbour of BRCA1 LncRNA 2 (NBR2) and Copine 1 (CPNE1) and age at menopause and expression of Leucine Rich Repeat Containing 37A (LRRC37A), Leucine Rich Repeat Containing 37 Member A2 (LRRC37A2) and Charged Multivesicular Body Protein 4C (CHMP4C) and epithelial ovarian cancer (7).

Other TWAS methods predict genome-wide expression into a GWAS dataset using the weighted effect of each SNP on each cis-gene from a reference set and then test the association between levels of expression and the trait (91). A TWAS performed using estimates of the genetic effects of gene expression in endometrium and endometriosis GWA summary statistics identified 252 genes in 33 loci associated with endometriosis including 28 loci that had not previously been identified as genome-wide significant (7). Many loci identified by the TWAS contained several genes whose expression was correlated highlighting that not all risk loci may have a single target gene. This was also shown in a recent analysis of the chromosome 6q25 risk locus near *ESR1* which showed that expression of genes in this region were highly correlated and that these genes are likely co-regulated (22). This approach has the potential to identify target genes for a range of other endometrial pathologies and fertility traits for which GWA summary statistics are available.

To better understand the mechanisms by which genetic variants are effecting gene expression and disease, analytic approaches have been developed to integrate GWAS and eQTL data with other molecular traits including protein expression, splicing, methylation and various epigenic marks (94). Risk variants associated with both expression and disease can also be functionally annotated using epigenic databases, such as EpiMap (95), RoadMap (96) and ENCODE (97) however, these databases lack epigenetic data from endometrial relevant tissues and cell-types. Previous investigations of the interactions between endometriosis risk SNPs in the 1p36.12 locus and candidate target genes using chromosome conformation capture (3C) in Ishikawa cell lines suggest that endometriosis risk SNPs interact with the promoters of both *LINC00339* and *CDC42*. Subsequent luciferase reporter assays suggested the risk SNP rs12038474 was located in a transcriptional silencer for *CDC42* and the risk allele increases expression of *CDC42* in blood (93). More recently, promoter associated chromatin looping from HiChIP analysis in an endometrial cancer cell line provided evidence of an interaction between a variant in the 1p36.12 locus associated with endometrial cancer, endometriosis and pelvic organ prolapse, and promoter regions of *CDC42* and *WNT4* (98). Expression of *LINC00339* has also been reported in endometriotic lesions and perturbation of *LINC00339* expression in endometrial stromal cells was shown to alter expression of genes in immune defence pathways (99).

Identification of genes associated with disease risk in endometrium can also provide insight into putative pathogenetic pathways that can be targeted for disease prevention, management and treatment. Genes functionally annotated to endometriosis risk loci have roles in hormone metabolism, cell cycle regulation, proliferation and adhesion (92). Oestrogen-responsive growth regulation by oestrogen in breast cancer 1 (*GREB1*) is an essential component of the oestrogen receptor transcription complex (100), risk variants have been associated with *GREB1* splicing, epigenic regulation and TF binding sites (8, 101). Risk variants for endometriosis on chromosome 6p25.1 are located in regulatory regions near oestrogen receptor 1 (*ESR1)* and both VEZT and FGD6 on chromosome 12q22 have roles in cell adhesion (102, 103). *CDC42* is involved in cell cycle regulation with evidence suggesting that *FGD6* activates *CDC42* to coordinate cell adhesion (104). Genetic regulation of genes involved in maintaining the endometrial environment via regulation of cell proliferation and immune response (*PAEP, SPP1, IL15, TSPAN8, OLFM1, MMP7* and *CXXC1*) have also been associated with endometrial receptivity, fecundity and implantation failure (6, 105).

Formal overlap between GWAS signals and eQTLs identifies some likely candidate genes, but the proportion of clear relationships between genetic risk factors and functional candidates has been disappointing. There are several possible explanations. As noted above, a high proportion of eQTLs for the small endometrial studies, and in GTEx, are common to many tissues (6, 7, 33). Some argue that eQTLs of large effect, and common to many tissues, may be neutral or have limited functional effects under steady state conditions (106). These eQTLs are rarely associated with genes that are intolerant to loss of function mutations (10, 106). Consequently, genes regulated by these eQTLs can vary in expression level, or in protein-coding sequence, with limited functional effects and are therefore not subject to negative genetic selection. Umans et al. (106) suggest we may have more success using a dynamic regulatory approach mapping eQTLs in model systems subject to experimental stimulation. This will uncover tissue or cell specific eQTLs more likely to have functional effects associated with disease risk. Studies in endometrium may satisfy this experimental approach because of the highly variable gene expression across the menstrual cycle (6, 7). While our studies are still relatively small, we found few context-specific eQTL, where genetic effects on gene expression were identified at only one stage of the menstrual cycle and we did not see evidence to support the dynamic regulatory approach.

It is estimated that, compared to *cis*-eQTLs, *trans*-eQTLs explain the majority of heritability in gene expression (10, 107). The large eQTLGen study had greater power to detect *trans*-eQTL and tested overlap between 10,317 GWAS signals for complex traits and *trans*-eQTLs. A high proportion (37%) of GWAS signals were associated with *trans*-eQTL effects. *Trans*-eQTLs are considered to be more specific for individual tissues and cell types and analysis of available single-cell data sets for blood showed nominal replication of 84% of the disease associated *trans*-eQTL effects (10). Taken together, results suggest we need to identify more tissue and cell-type specific *cis*- and *trans*-eQTLs and splice variant QTLs to understand how genetic risk factors change gene regulation and increase disease risk.

## Summary and Conclusions

Gene expression is influenced by the external and internal environment through neuronal, hormonal and other signalling pathways and by genetic and epigenetic factors. In addition to the dynamic cyclical changes in cellular structure and function, the human endometrium has complex mechanisms regulating gene transcription. Understanding and controlling for sources of variation in endometrial transcription is critical. Critical sources of variation include accurate determination of stage of the menstrual cycle, genetic effects on expression level and splicing and variable cell composition and heterogeniety.

Genome-wide gene expression studies have characterised significant changes in endometrial gene expression and activation of genes, across the menstrual cycle. These strong effects of menstrual cycle stage on transcription in the endometrium likely reflect changes in cell composition and response to circulating steroid hormones. Independent of menstrual cycle phase, genetic variation between individuals is also associated with the level of expression of >600 genes in endometrium of which the majority of effects are shared between tissues and are most highly correlated with biologically similar tissues. Epigenomic analyses in endometrium indicate transcriptional variation can also be mediated by genetic regulation of methylation. The ability to identify more subtle, tissue-specific, genetic effects on regulation is limited by the power and size of studies, sample heterogeneity and context.

Endometrium is made up of multiple cell types and changes in cell composition and activity change across the menstrual cycle. Understanding cell-type specific genetic effects on gene expression is challenging and functional effects may require intercellular communication between more than one cell types. Methods of cell-type deconvolution and single-cell sequencing are being adapted and applied to studies in endometrium to investigate cell-type specific regulation.

Evidence suggests that genetic regulation of endometrial gene expression (eQTL) contributes to reproductive traits and diseases. Effects of genetic variation on RNA splicing (sQTL) and distal genes (*trans*-eQTL), and cell-type specific genetic effects, are also enriched for variants associated with complex traits and diseases highlighting new directions for investigation. Understanding which genes and pathways should be targeted in which cell type can be used to improve fertility and disease management.

A comprehensive understanding of factors affecting regulation of transcription in the endometrium and endometrial cell-types is vital for accurate analysis and interpretation of data from endometrium across biological and disease contexts. Hormonal, genetic, epigenetic and cell-type specific regulation of gene expression can influence menstruation, fertility and endometrial pathologies making it vital for researchers and clinicians to consider an individual's genetic background and hormonal influences when investigating, assessing and managing fertility and disease.

## Author Contributions

All authors listed have made a substantial, direct, and intellectual contribution to the work and approved it for publication.

## Funding

This work was supported by the National Health and Medical Research Council of Australia (grants GNT1026033, GNT1049472, GNT1105321, GNT1078399, GNT1147846, and GNT1177194) and Medical Research Future Fund Research (Grant MRF1199785).

## Conflict of Interest

The authors declare that the research was conducted in the absence of any commercial or financial relationships that could be construed as a potential conflict of interest.

## Publisher's Note

All claims expressed in this article are solely those of the authors and do not necessarily represent those of their affiliated organizations, or those of the publisher, the editors and the reviewers. Any product that may be evaluated in this article, or claim that may be made by its manufacturer, is not guaranteed or endorsed by the publisher.
